# Is Fecal Leukocyte Test a good predictor of *Clostridium difficile *associated diarrhea?

**DOI:** 10.1186/1476-0711-5-9

**Published:** 2006-04-19

**Authors:** Savio Reddymasu, Ankur Sheth, Daniel E Banks

**Affiliations:** 1 Department of Medicine, Louisiana State University Health Sciences Center, Shreveport, LA, USA

## Abstract

**Background:**

Fecal leukocyte test (FLT) is widely used to screen for invasive diarrheas including *C. difficile *associated diarrhea (CDAD), which account for more than 25 % of all antibiotic associated diarrhea.

**Method:**

263 stool samples from patients with suspected CDAD were studied simultaneously for fecal leukocyte test (FLT) and Clostridium difficile toxin assay (CDTA). FLT was performed by the Giemsa technique and CDTA was performed by enzyme immuno assay (EIA).

**Results:**

Sensitivity, specificity, positive predictive value and negative predictive value of FLT as compared to CDTA were 30%, 74.9%, 13.2% and 89.3% respectively.

**Conclusion:**

Considering the poor sensitivity of FLT, and the comparable cost and time of obtaining a CDTA at our institution, we conclude that FLT is not a good screening test for CDAD. Possible reasons for FLT being a poor predictor of CDTA are discussed.

## Introduction

Willmore and Shearman[1] first described the fecal leukocyte stain in 1918 followed by its clinical use for diagnosis of bacterial diarrhea in 1972 by Harris *et al *[2]. Today fecal leukocyte testing (FLT) is widely used to screen for inflammatory diarrhea including *C. difficile *diarrhea, which account for more than 25 % of all antibiotic associated diarrhea. Laboratory diagnosis of *C. difficile *associated diarrhea (CDAD) is based on the detection of *C. difficile *toxins in stool samples by a cell culture cytotoxicity assay or enzyme immunoassay. We evaluated FLT within an inpatient cohort, defining the test's sensitivity, specificity, positive predictive value (PPV) and negative predictive value (NPV) for patients with CDAD.

## Materials and methods

After obtaining approval from the institutional review board at Louisiana State University Health Sciences Center, Shreveport, a retrospective review of laboratory data was done on 263 inpatients whose stool samples were simultaneously submitted for FLT and *C. difficile *toxin assay (CDTA). Specimens were submitted between January 2001 and June 2004.

FLT was performed on fresh stool specimens. Samples are obtained in a clean dry container. Stool specimens are applied as a thin smear on a slide using a cotton swab. After the slides are air dried, they are smeared with Wright stain and examined under the microscope for white blood cells (WBC). Criteria for positive FLT are > 1 WBC/high power field. The cost of obtaining a FLT is about $30. Time of availability of the test is approximately one hour after submission of the stool specimen. Specimens were processed in the same laboratory, but likely by different technicians.

CDTA was done using Premier™ Toxins A&B (PT A&B). PT A&B is an EIA for the direct detection of C.*difficile *toxin A and toxin B. CDAD was defined as diarrhea with a positive CDTA. The cost of obtaining CDTA is about $30. Time of availability is approximately 45 minutes after submission of the specimen.

## Results

Of the 263 stool samples tested at the same time for FLT and CDTA, 68 were positive for FLT and 30 were positive for CDTA (Table 1). The sensitivity and specificity of FLT as compared to CDTA was 30 % and 74.9 % respectively. The PPV and NPV of FLT was 13.2 % and 89.3 % respectively for CDTA. 70% of all stool specimens positive for CDTA had a negative FLT. Prevalence of a positive CDTA was 11.4%.

**Table 1:** Comparison of result from FLT and EIA for *C. difficile *toxins.

Test	*EIA for *C.difficile *toxins*			Total
	
Fecal Leukocyte Test		Positive	Negative	
	
	Positive	9	59	68
	Negative	21	174	195
	Total	30	233	263

## Discussion

Clostridium difficile (*C.difficile*) is the commonest cause of nosocomial diarrhea associated with significant morbidity and health care costs.[3,4]. Despite being perceived as a common cause of antibiotic associated diarrhea, *C.difficile *associated diarrhea (CDAD) is often difficult to diagnose.[3] Enzyme immunoassay (EIA) against *C.difficile *toxin A and/or toxin B in stool specimens is currently the acceptable method of diagnosing C.difficile diarrhea. [5-7] Since C.difficile is considered to be a type of invasive diarrhea, fecal leukocyte test (FLT), that has been proposed by Harris et al [2] as a rapid test to differentiate invasive versus non-invasive diarrhea [2,8] might be useful as a predictor of C.difficile diarrhea.

There is conflicting evidence regarding the use of FLT as a screening test for CDAD. While the studies done by Savola et al [9], Mark et al [10], Manabe et al [11] and Shanholtzer et al [12] concluded FLT as a poor predictor of CDAD; studies done by Bartlett et al [13] and Fekety et al [14] proposed that FLT might be a useful predictor in CDAD. The results of our study reinforce the fact that FLT is a poor predictor of CDAD; because 70% of stool specimens positive for *C. difficile *toxin are negative for fecal leukocyte. There is no significant time or cost savings by obtaining a FLT, as opposed to CDTA in patients with suspected CDAD.

Poor predictability of CDAD with a FLT can partially be explained by inter-observer variability in interpreting fecal leukocytes under microscopy. False negative results could also be due to degeneration of fecal leukocytes secondary to delay in processing stool specimens. This is reinforced by the fact that objective tests to detect fecal leukocytes like the fecal lactoferrin [15-18] or the fecal leukocyte esterase test [19] are better indicators of fecal leukocytes than the FLT. Low PPV can also be explained by the fact that EIA for toxin detection is less sensitive compared to the neutralizing tissue culture cytotoxicity assay for CDAD.

Considering the poor sensitivity and comparable cost and promptness with *C.difficile *toxins assay in our institution, we conclude that FLT is not a good screening test for CDAD in inpatients.

## Financial disclosure

None

## Competing interests

The author(s) declare that they have no competing interests.

## Authors' contributions

SR conceived of the study and participated in its design and data collection. AR participated in data collection, analysis and drafting of manuscript. DB participated in design and coordination of the study. All authors read and approved the final version of the manuscript
